# UVB irradiation differential regulate miRNAs expression in skin photoaging^☆^

**DOI:** 10.1016/j.abd.2022.01.003

**Published:** 2022-05-31

**Authors:** Yuan Fang, Lei Chen, Xin Wang, Xu Li, Wu Xiong, Xi Zhang, Yufang Zhang, Lu Han, Ke Cao, Xiang Chen, Haibo Li, Jianda Zhou

**Affiliations:** a Department of Plastic Surgery, The Third Xiangya Hospital, Central South University, Changsha, Hunan, China; b Department of Plastic and Reconstructive Surgery, Shanghai Ninth People's Hospital, Shanghai Jiao Tong University School of Medicine, Shanghai, China; c Second Xiangya Hospital, Central South University, Changsha, Hunan, China; d First Affiliated Hospital of Hunan University of Chinese Medicine, Changsha, China; e Hunan Brain Hospital, Clinical Medical School of Hunan University of Chinese Medicine, Changsha, Hunan, China; f Anyang Tumor Hospital, The Fourth Affiliated Hospital of Henan University of Science and Technology, Anyang, Hunan, China; g Neihuang Chinese Medicine Hospital, Anyang, Henan, China; h Department of Dermatology, Xiangya Hospital, Central South University, Changsha, Hunan, China

**Keywords:** Computational biology, MicroRNAs, Skin aging, Ultraviolet rays

## Abstract

**Background:**

UVB irradiation can cause acute damage such as sunburn, or photoaging and melanoma, all of which are major health threats.

**Objective:**

This study was designed to investigate the mechanism of skin photoaging induced by UVB radiation in mice through the analysis of the differential expression of miRNAs.

**Methods:**

A UVB irradiation photoaging model was constructed. HE and Masson special stains were used to examine the modifications in the epidermis and dermis of mice. The miRNA expression profiles of the mouse skin model exposed to UVB radiation and the normal skin of mice were analyzed using miRNA-sequence analysis. GO and Pathway analysis were employed for the prediction of miRNA targets.

**Results:**

A total of 23 miRNAs were evaluated for significantly different expressions in comparison to normal skin. Among them, 7 miRNAs were up-regulated and 16 were down-regulated in the skin with photoaging of mice exposed to UVB irradiation. The differential expression of miRNA is related to a variety of signal transduction pathways, among which mmu-miR-195a-5p and mitogen-activated protein kinase (MAPK) signal pathways are crucial. There was a significant differential expression of miRNA in the skin of normal mice in comparison with the skin with photoaging induced by UVB irradiation.

**Study limitations:**

Due to time and energy constraints, the specific protein level verification, MAPK pathway exploration, and miR-195a-5p downstream molecular mechanism need to be further studied in the future.

**Conclusions:**

UVB-induced skin photoaging can be diagnosed and treated using miRNA.

## Introduction

Solar ultraviolet radiation is a major environmental carcinogen. There were three regions according to the different wavelengths. Short wave UVC is 200‒280 nm, mediumwave UVB is 280‒320 nm, and longwave UVA is 320‒400 nm.^1^, ^2^ UVB has two sides. According to different irradiation doses, different disease models will have different effects. Many researchers use UVB to treat human diseases, such as vitiligo.^3^ The model the authors used in this article is the mice skin photoaging model. UVB irradiation can cause acute damage such as sunburn, photoaging, and melanoma, all of which are major health threats. Due to its short wavelength and strong intensity, UVB (290‒320 nm) causes significant damage to the epidermis. Keratinocytes were its target cells, which could induce adverse skin reactions such as edema, erythema, blisters, skin pigmentation, and premature photoaging.^4^, ^5^ If these lesions are not repaired in time, severe structural distortion of DNA molecules may be caused by destroying cell vitality and affecting its function, and even lead to skin aging and even skin cancer.^6^

MicroRNAs (miRNAs) are considered a group of small endogenous non-coding RNAs (about 23 nucleotides) that are associated with post-transcriptional gene suppression.^7^, ^8^ miRNAs mainly act on post-transcriptional regulation and are important in maintaining normal cell homeostasis.^9^ Several studies indicate that miRNAs have a role in a variety of biological processes, including development, differentiation, reproduction, and apoptosis. Recently, researchers have found a relationship between miRNA and the occurrence and progression of some skin diseases, such as miRNAs and skin cancers, pigmentation, inflammatory diseases, autoimmune diseases, wound healing, and photoaging.^10^, ^11^, ^12^, ^13^ Accounted for 1%∼5% of the human genome miRNAs have about 30% of protein-coding genes regulating,^14^ and its importance to the whole body is obvious. Likewise, the mechanism of skin photoaging mediated by UVB exposure remains unknown further research can determine the mechanical relationship between miRNAs and UVB irradiation, and it will aid in the prevention of such injury as well as give diagnostic biomarkers and novel nucleic acid targets for treatment. The differential expression of miRNAs in skin photoaging was searched by UVB radiation and normal skin tissue by using miRNAs sequence detection technology to explore the mechanism of skin photoaging caused by UVB.

## Method and material

### Animal models

Twelve adult male mice with C57BL/6 were selected for this study. These mice originate from the experimental Animal Center of Third Xiangya Hospital, Central South University, China. The mice were aged about 8 weeks old and weighed 25–30 g. Mice were kept in a temperature and humidity-controlled facility with enough food and water. All animal feeding procedures were performed following the National Institutes of Health's Guidelines for the Care and Use of Laboratory Animals. Central South University's Bioethics Committee authorized all animal experiment procedures.

### Ultraviolet radiation

The mice were randomly divided into three experimental groups and one control group, which were control group A (with a radiance of 0 mJ/cm^2^), UVB irradiation group B (with a radiance of 90 mJ/cm^2^), UVB irradiation group C (with the radiance of 180 mJ/cm^2^), and UVB irradiation group D (with the radiance of 360 mJ/cm^2^). The mice were first sedated (intraperitoneal injection of sodium pentobarbital), and then their backs were depilated. The experiment was carried out using the UVB (290–320 nm) spectrum of the SS-03BUltraviolet Phototherapy Instrument. The mice in groups A, B, C, and D were irradiated at the dose of 0, 90, 180, and 360 mJ/m^2^ respectively. The modifications in the mouse's back skin were continuously monitored and photographed during the experiment. On the ninth day, the mice were killed, and the skin tissues were collected.

### Skin histological staining

On day 9^th^, skin tissue was taken. These samples were fixed in 0.1 M phosphate buffer (PB, Ph 7.4) with 4% paraformaldehyde for 24 hours. The tissues were embedded in paraffin. Sample sections were prepared (5 μm). Hematoxylin staining of the nuclei and cytoplasmic eosin staining of the cytoplasm was done. The photographs were taken and analyzed after wiping with a microscope.

### Library preparation

The library was prepared using the NEBNext® Multiplex Small RNA Library Prep Set for Illumina® and the nanodropND-1000 Agilent 2100 Bioanalyzer.

Agarose electrophoresis was used to check the integrity of the RNA, which was then quantified on the NanoDrop ND-1000 instrument. To remove RNA modifications that interfere with small RNA-seq library construction, the total RNA samples underwent 3’-aminoacyl (charged) deacylation to 3’-OH for 3’-adaptor ligation, 3’-cP ('2',3’-cyclic phosphate) removal to 3’-OH for 3’-adaptor ligations, 5’-OH (hydroxyl group) phosphorylation to 5’-P for 5’-adaptor ligations, and m1A and m3C demethylation for efficient reverse transcription. For miRNA-seq library preparation, each sample's total RNA was pretreated. The library preparation procedures included: 3’-adapter ligation, 5’-adapter ligation, cDNA synthesis, Polymerase Chain Reaction (PCR) amplification, size selection of ∼134–160 bp PCR amplified fragments (corresponding to ∼14–40 nt small RNAs). The Agilent 2100 Bioanalyzer was used to quantify the completed libraries. Based on the quantification results, the libraries were combined in equal proportions and used for sequencing.

### Sequencing

The DNA fragments from the well-mixed libraries were denatured with 0.1 M NaOH to generate single-stranded DNA molecules and loaded onto the reagent cartridge at 1.8 pM. The sequencing runs were performed on the Illumina NextSeq 500 system using the NextSeq 500/550 V2 kit (#FC-404-2005, Illumina) according to the package recommendations. The sequencing was performed by running 50 cycles.

### Data analysis

**Figure 1:**
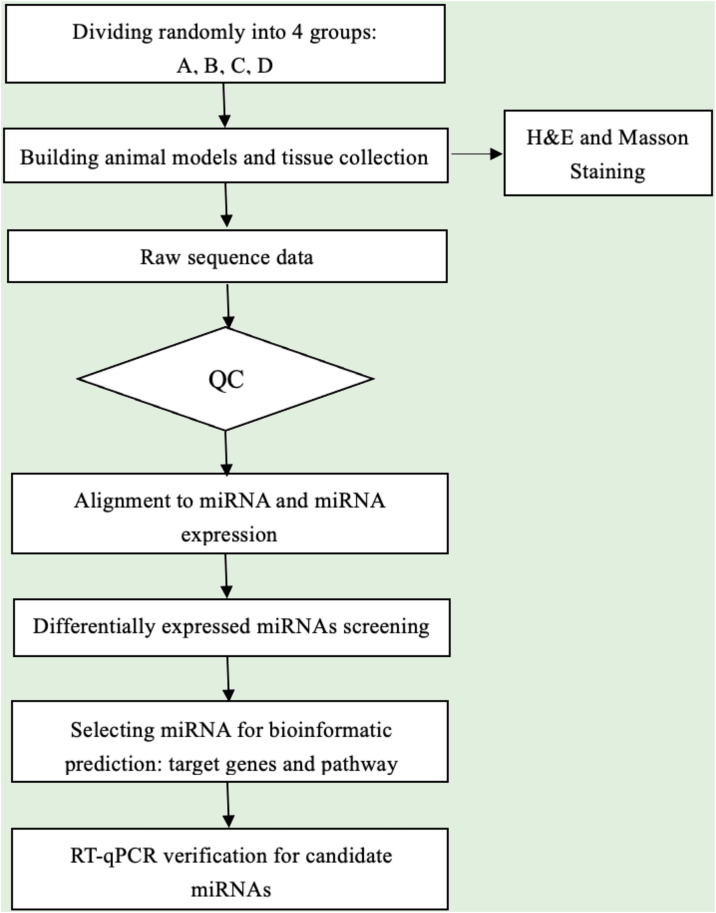
miRNA-seq experiment workflow.

Sequence quality was examined via FastQC, and trimmed reads were aligned, which were allowed for one mismatch only. Afterward, align the unmapped reads, which were allowed for one mismatch only with bowtie software.^15^ Align the remaining read segments, allowing just one to mismatch with the miRNA reference sequence and miRDeep2.^16^ Based on the number of reads mapped, miRNA expression profiling may be estimated. The miRNAs differentially expressed are screened based on the count value with R package edgeR.^17^ miRNA-seq data analysis workflow is shown in Fig. 1. Figures were produced in R or Perl environment for statistical calculation and graphics of 'miRNAs' expression.

### Target gene prediction and function analysis

Past studies have suggested TargetScan that mirdbV5 predicts miRNA targets. Only genes that were predicted by both programs to be miRNA were accepted. Gene Ontology (GO) and Kyoto Encyclopedia of Gene and Genomes (KEGG) signal transduction pathway enrichment analyses were performed to predict functions of specific miRNA. The GO database was classified into three categories: biological process, cellular component, and molecular function. To assess the network of differentially expressed genes, the authors used KEGG, a pathway analysis reference database. The meaning of GO words and route terms was assessed using the p-value. The lower the p-value means a more important term (recommended p-value was ≤0.05).

### RT-qPCR

Design primers: the related primers were designed and synthesized by software in Table 1. Real-time quantitative PCR: the system was established for real-time quantitative analysis. All cDNA samples were configured with a Real-time PCR reaction system. Add the sample to each hole corresponding to the PCR plate. Place the PCR plate on the real-time PCR instrument for PCR reaction. Each sample's target genes and housekeeping genes were submitted to a real-time PCR reaction. The machine-generated the concentration findings of target genes and housekeeper genes in each sample based on the gradient dilution DNA standard curve. Relative miRNA levels were normalized according to U6 snRNA, and the mean and standard error were calculated.

**Table 1:** Sequences of primers for RT-qPCR.

mmu-miR-195a-5p		CGGTAGCAGCACAGAAATATTGGC
U6	F	CTCGCTTCGGCAGCACA
	R	AACGCTTCACGAATTTGCGT
		Product length 94 bp

### Statistical analysis

The data were presented as means ± standard deviations. miRNA expression followed a discrete distribution. Accordingly, the significant differences between groups were compared by a negative binomial distribution. The differentially expressed miRNAs were then screened. The recommended p-value was ≤0.05.

## Results

### UVB-induced skin tissue damage

**Figure 2:**
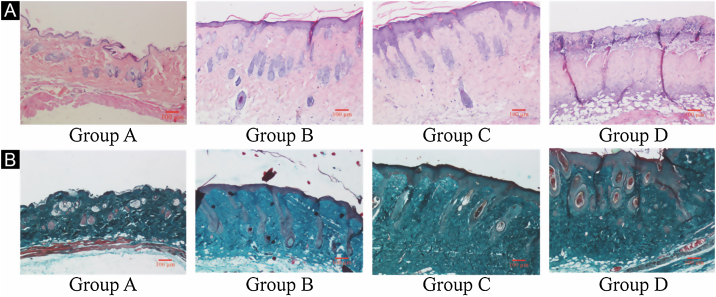
Histological changes of mouse back skin under different doses of UVB irradiation. See Figure A for HE is staining and Figure B for Masson staining. Radiation of Group A dose is 0 mJ/cm^2^; Radiation of Group B dose is 90 mJ/cm^2^; Radiation of Group C dose is 180 mJ/cm^2^; Radiation of Group D dose is 360 mJ/cm^2^. Scale bars are 100 µm.

H&E and Masson staining were exhibited in Fig. 2. Compared to the control group A, the epidermis layer of the experimental 'groups' B/C/D increased at different levels. Furthermore, the thickness increased with the increase in radiation dose. The dermis layer in control group A had a uniform distribution of fibrous tissue, whereas the dermis layer in experimental groups B/C/D showed a disordered fiber arrangement. Compared with control group A, experimental group B had mild epidermal thickening, dermal papillary layer, intact basement membrane, and a small amount of inflammatory cell infiltration, indicating that ultraviolet radiation had a damaging effect on the skin. Compared with experimental group B, the epidermis of experimental group C continues to thicken, and the dermal papilla layer could be seen, the basement membrane degradation and inflammatory cell infiltration could be seen. Compared with control group A, the thickness of the skin epidermis in experimental group D was significantly different, and even some areas of skin necrosis and falling off, the arrangement of dermis tissue was disordered, the basement membrane degradation and inflammatory cell infiltration could be seen.

### Screening of differentially expressed miRNA

**Figure 3:**
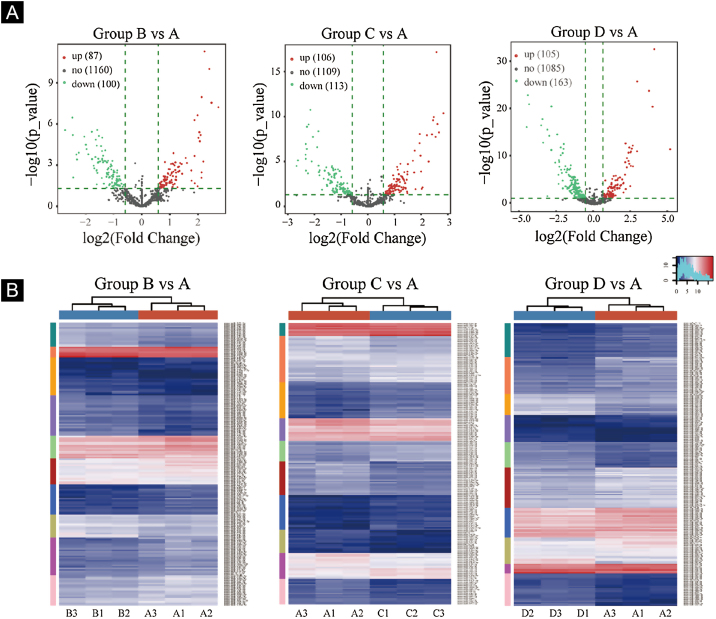
Differentially expressed miRNA screening. (A), The volcano plots. Red circles indicate statistically up-regulated expression, green circles indicate down-regulated, and grey circles indicate non-differentially expressed miRNAs. (B), The hierarchical clustering heatmap for miRNAs. The color scale is show below: blue represents an expression level below the mean, and red represents an expression lever above the mean.

Volcano plots were plotted based on miRNA with CPM values (see Fig. 3). According to these volcano plots, the authors could find the differential expression of miRNAs. There were 87 up-regulated miRNAs and 100 downregulated miRNAs in Fig. 3A (B vs. A). In Fig. 3B (C vs. A), there were 106 up-regulated miRNA and 113 downregulated miRNAs. In Fig. 3C (D vs. A), there were 105 up-regulated miRNA and 163 downregulated miRNAs.

Hierarchical clustering using differentially expressed miRNAs is shown in Fig. 3. Each row represented a different miRNA, and each column represented a different sample. The findings of hierarchical clustering revealed that there were differences in the expression of miRNAs between samples. The authors screened 7 significantly up-regulated miRNAs and 16 significantly down-regulated miRNAs in groups B vs. A, C vs. A, and D vs. A (see Fig. 3D/E/F).

First, standardize the original data. Then, 23 differentially expressed miRNAs were exposed by miRNA sequencing results. Compared with the Ctrl-A group, 16 miRNAs were downregulated, and 7 miRNAs were up-regulated in the irradiated mice group (Supplementary Tables 1 and 2).

### Target genes prediction

**Figure 4:**
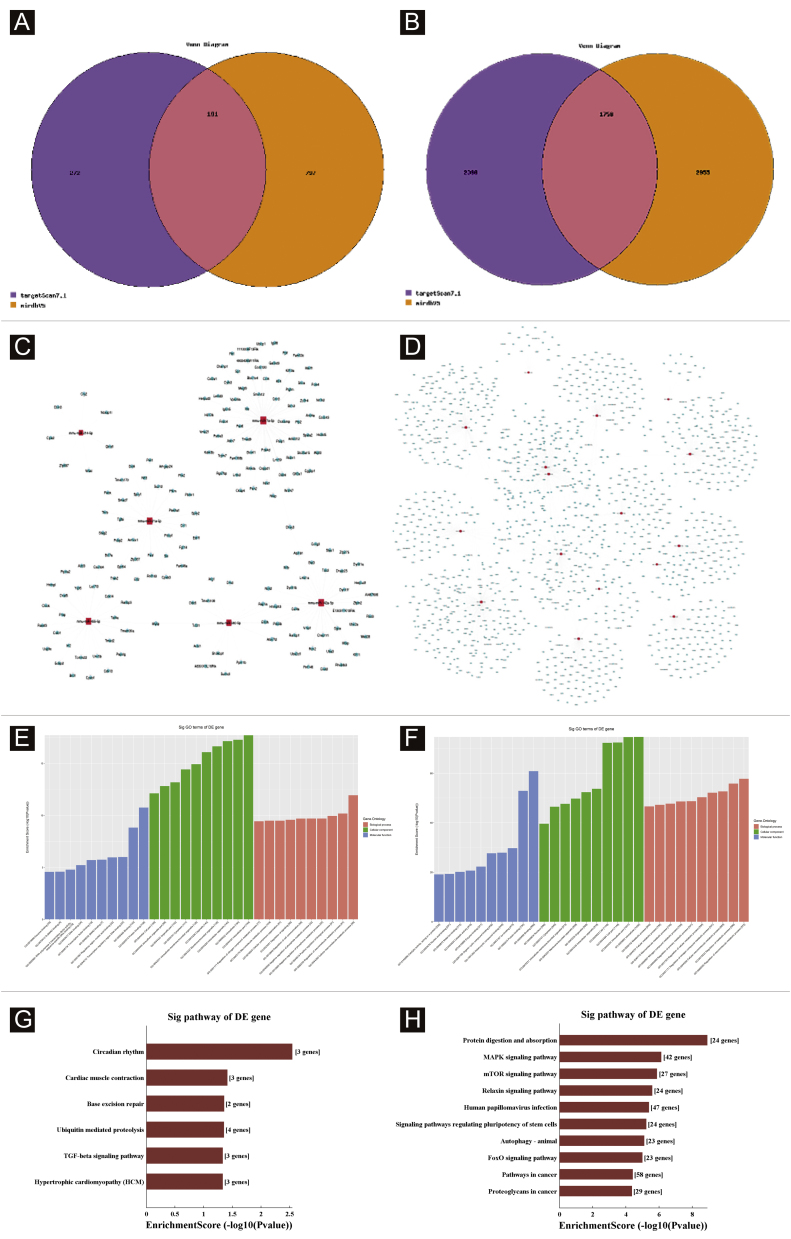
Bioinformatic Prediction. (A-B), Venn diagram. (C), The up-regulated miRNA network analysis. (D), The downregulated miRNA network analysis. The general GO annotations for cellular component, molecular function, and biological processes of the target genes regulated by the up-regulated miRNAs (E) and downregulated miRNAs (F). KEGG pathway analysis of the target genes regulated by the up-regulated miRNAs (G) and downregulated miRNAs (H).

Targetscan7.1 and mirdbV5wereused to predict 'miRNAs' target genes. Targetscan7.1: (from http://www.targetscan.org/mmu_71/). MirdbV5: (from http://mirdb.org/miRDB/.Targetscan7.1 identified 463 target genes in the 7 up-regulated miRNAs; mirdbV5 predicted 988 target genes, and the intersection of two target genes was 191, as shown by the Venn diagram (Fig. 4A). In the 16 down-regulated miRNAs, targetscan7.1 predicted 4146 target genes, mirdbV5 predicted 4711 target genes, and the intersection of two target genes was 1756, which were reflected by the Venn diagram (Fig. 4B). Using the 191 up-regulated target genes and 1,756 down-regulated target genes that the authors predicted, the authors developed a network diagram (Fig. 4C-D) to show the relationship between miRNAs and target genes. The blue circular nodes in the following network represent mRNAs, whereas the red rectangle nodes represent miRNAs. It is necessary to predict the target of the miRNA regulatory gene to further understand the miRNA function. Through GO analysis, the authors have detected the GO terms to predict the function of these target genes. Fig. 4E shows all of the up-regulated miRNA target genes, as well as the top ten GO terms with the highest enrichment. Similarly, the target genes for miRNA with a downregulation trend are listed in Fig. 4F. According to bioinformatics analysis, the authors believe that the predicted target gene function of up-regulating miRNA is mainly concentrated in the intracellular part, cellular macromolecule metabolic process, and protein binding. The target gene's function in downregulating miRNA is primarily focused on intracellular binding and control of the macromolecule metabolic process. The gene was shown to be a component of the KEGG signaling pathway. Through Pathway analysis, the authors screened out some signal pathways closely related to UV-induced acute skin injury. According to the findings, the most prevalent genes in the down-regulation genes were those involved in cancer, human papillomavirus infection, and the MAPK signaling system (see Fig. 4G-H). The authors chose the MAPK signaling pathway for further study because the first two pathways had minimal correlation with the acute injury the authors evaluated.

### MAPK signaling pathway

**Figure 5:**
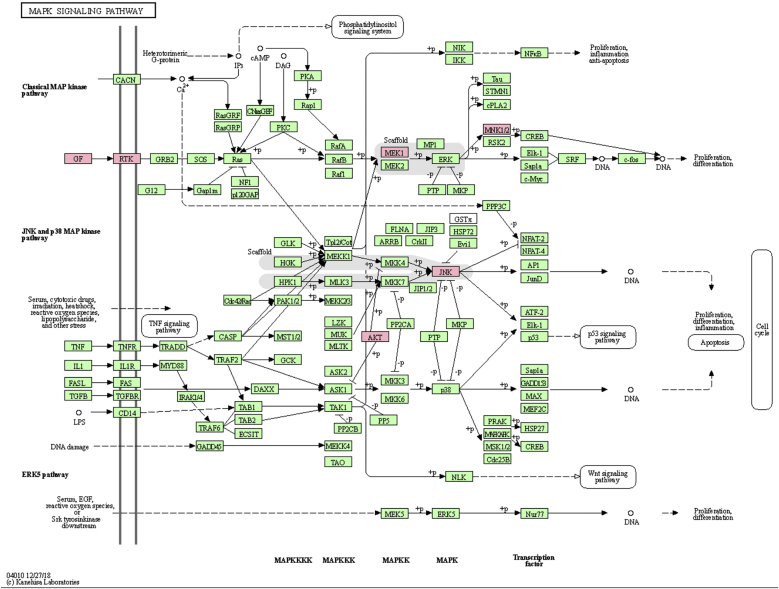
MAPK Signaling Pathway.

MAPK pathway is a common pathway in the human body which is related to stress and other complex physiological processes. Radiation, osmotic pressure changes, growth factors, and cytokines are all instances of stimuli that might activate the MAPK signal transduction pathway. The five important target genes predicted by miR-195a-5p were all located in the MAPK signaling pathway; they were AKT(AKT323797), RTK (INSR 16337), JNK (MAPK8 26419), MEK1(MAP2K1 26395), MNK1/2(MKNK1 17346), GF (VEGFA 22339) (see Fig. 5). It was suggested that ultraviolet radiation could induce acute skin injury by down-regulating miR-195a-5p and activating MAPK signaling pathway.

### PT-qPCR verification

**Figure 6:**
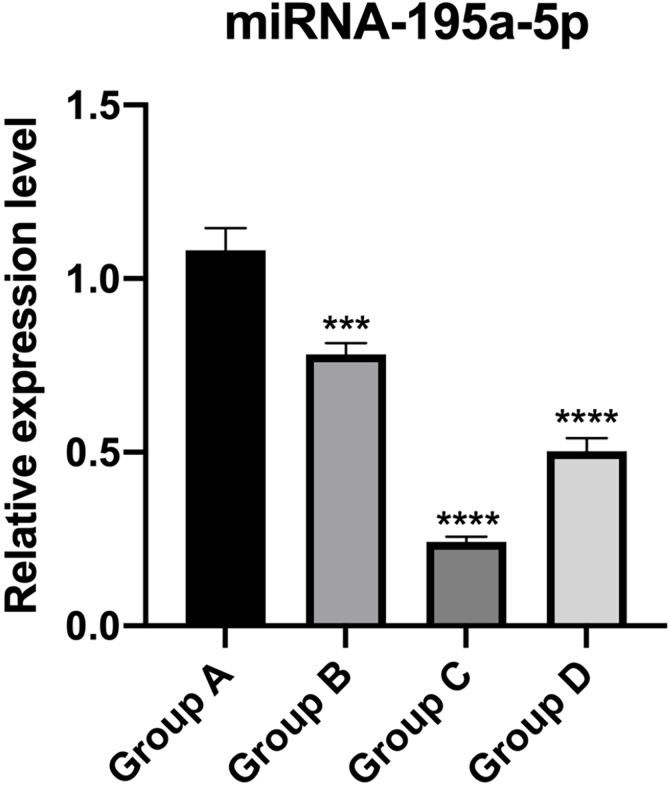
Validation of miRNA-195a-5p using RT-qPCR. The data were normalized using the mean ± Standard Error of the Mean (SEM). ∗∗∗p ≤ 0.001, ∗∗∗∗p ≤ 0.0001.

The relative expression of miR-195a-5p in several experimental groups was statistically examined. It can be seen from Fig. 6 that the expression of miR-195a-5p in the three experimental groups showed a downward trend after UV irradiation, which was in accord with the prediction of miR-195a-5p. Therefore, miR-195a-5p was selected for further study.

## Discussion

Solar UV radiation is a significant carcinogen in the environment. UV radiation's clinical effect on normal skin might be acute or chronic. Acute effects mainly include erythema (sunburn), pigmentation (tanning), acquired immunosuppression, and innate immune enhancement, which are mainly caused by UVB.^18^, ^19^ More than 80% of the human body is suffering from skin photoaging caused by UV radiation due to the loss of the ozone layer caused by environmental pollution, excessive sunbathing, and other factors.^20^ One of the research hotspots is the prevention and treatment of UV radiation damage. The authors’ study group has a research foundation that reveals that UV radiation-induced photoaging is related to the ERK/MAPK pathway.^21^, ^22^ Although UV is a stimulator that can activate multiple signal cascades, these signals cascades activation and their role in the MAPK pathway are still unclear.

It has been reported that many miRNAs are closely related to skin-related diseases, such as Systemic Lupus Erythematosus (SLE), an immune-related disease.^23^ miR-377 can directly target and inhibit DNMT1, thereby methylating p53 and regulating the aging of human skin fibroblasts.^24^ Further research can identify the relationship between miRNA and the UVB UVB-induced skin photoaging process; efficient preventive measures, as well as biomarkers for disease diagnosis and novel nucleic acid targets for therapy, would be feasible. According to the results of the miRNA Sequence, the differentially expressed miRNAs were screened. There were 7 up-regulated miRNAs: mmu-miR-21a-5p, mmu-miR-214-5p, mmu-miR-126a-3p, mmu-miR-142a-5p, mmu-miR-7a-5p, mmu-miR-455-5p and mmu-miR-340-5p. 16 miRNAs that were significantly down-regulated are screened out, including mmu-miR-200a-3p, mmu-miR-324-5p, mmu-miR-133b-3p, mmu-miR-133a-3p, mmu-miR-101a-3p, mmu-miR-101c, mmu-miR-195a-5p, mmu-let-7b-5p, mmu-miR-199b-5p, mmu-miR-10a-5p, mmu-miR-30b-5p, mmu-miR-26a-5p, mmu-miR-29a-3p, mmu-miR-188b-5p, mmu-miR-23a-3p. Sequencing results need to be verified by PT-qPCR to confirm the reliability of data and results. Given The authors’ previous research on mmu-miR-195a-5p in many aspects, mmu-miR-195a-5p for verification was selected. The expression of mmu-miR-195a-5p was down-regulated, which was consistent with the sequencing results, suggesting that the miRNA-seq data were true and reliable.

miR-195 is one of the key members of the micro-15/16/195/424/497 family, which can be activated in many diseases, such as cancer, hemangioma, and neuropathic pain.^25^, ^26^, ^27^ The mechanism of miR-195 mediated its action is extremely complex and involves various signal transduction pathways, such as Rb-E2F, PI3K/AKT, NF-kB, MAPK/REK, Wnt/ β -Catenin and so on.^28^, ^29^ This also confirms the findings of The present study’s KEGG pathway study. The authors hypothesized that miR-195a-5p might control the MAPK pathway. The five important target genes predicted by miR-195a-5p were all located in the MAPK signaling pathway; they are Akt (akt323797), RTK (INSR 16337), JNK (MAPK8 26419), MEK1 (MAP2K1 26395), MNK1/2 (MKNK1 17346), GF (VEGFA 22339). It was suggested that ultraviolet radiation could induce skin photoaging by down-regulating miR-195a-5p and activating MAPK signaling pathway.

There are still many limitations to this experiment. A cell model of UV-induced keratinocytes damage was established, observed a series of related biological expression changes, verified its function, and further explored the specific role of miR-195a-5p. The above analysis of the miR-195a-5p downstream molecular mechanism is only speculation but has not been confirmed. This experiment only validated the gene-level bioinformatics prediction. Gene transcription and translation to protein expression is a complicated process that must be validated at the protein level using techniques such as Western Blot or Immunohistochemistry. Due to time and energy constraints, the specific protein level verification, MAPK pathway exploration, and miR-195a-5p downstream molecular mechanism need to be further studied in the future.

## Conclusion

The present study established the relationship between miRNAs and UVB irradiation. These findings might help in preventing and delaying the occurrence of skin aging. Moreover, it can identify biomarkers for disease diagnosis as well as novel targets for treatment.

## Financial support

This work was supported by a grant from the National Major Science and Technology Projects of China (2018ZX10303502) and the Natural Science Foundation of China (82002065).

## Authors' contributions

Yuan Fang: Approval of the final version of the manuscript; Data collection, analysis, and interpretation; Effective participation in research orientation; Intellectual participation in propaedeutic and/or therapeutic management of studied cases; Manuscript critical review; Preparation and writing of the manuscript; Statistical analysis; Study conception and planning.

Lei Chen: Approval of the final version of the manuscript; Manuscript critical review.

Xin Wang: Approval of the final version of the manuscript; Manuscript critical review.

Xu Li: Approval of the final version of the manuscript; Data collection, analysis, and interpretation; Effective participation in research orientation; Intellectual participation in propaedeutic and/or therapeutic management of studied cases.

Wu Xiong: Approval of the final version of the manuscript; Manuscript critical review.

Xi Zhang: Approval of the final version of the manuscript; Manuscript critical review.

Yufang Zhang: Approval of the final version of the manuscript; Effective participation in research orientation; Manuscript critical review.

Lu Han: Approval of the final version of the manuscript; Manuscript critical review.

Ke Cao: Approval of the final version of the manuscript; Manuscript critical review.

Xiang Chen: Approval of the final version of the manuscript; Manuscript critical review.

Haibo Li: Approval of the final version of the manuscript; Statistical analysis; Manuscript critical review.

Jianda Zhou: Approval of the final version of the manuscript; Effective participation in research orientation; Manuscript critical review.

## Conflicts of interest

None declared.
