# Supplementary material for: UVB irradiation differential regulate miRNAs expression in skin photoaging
Source: An Bras Dermatol. 2022 May 31;97(4):458–66. doi: 10.1016/j.abd.2022.01.003 (PMC9263642; doi:10.1016/j.abd.2022.01.003)
Supplement: Supplementary file 1 [file mmc1.docx]

**Supplementary Table 1** miRNAs up-regulated.

| **microRNA** | **Group** | **B/C/D-CMP** | **A-CMP** | **Fold-Change** | **p-value** |
| --- | --- | --- | --- | --- | --- |
| mmu-miR-21a-5p | B vs. A | 16.69758523 | 14.46960099 | 4.684789554 | 5.02251E-12 |
|  | C vs. A | 17.04059892 | 14.47085308 | 5.937048235 | 6.76563E-18 |
|  | D vs. A | 17.3970933 | 14.53560424 | 7.267650575 | 1.83549E-26 |
| mmu-miR-214-5p | B vs. A | 8.080577856 | 7.211232339 | 1.826833964 | 0.027742406 |
|  | C vs. A | 8.430393442 | 7.195308118 | 2.353952687 | 0.000405572 |
|  | D vs. A | 8.514622963 | 7.28420605 | 2.346347853 | 0.000399519 |
| mmu-miR-126a-3p | B vs. A | 13.68083717 | 13.02695926 | 1.57339174 | 0.021982741 |
|  | C vs. A | 14.14741073 | 13.02670992 | 2.174525775 | 8.39786E-05 |
|  | D vs. A | 14.39563199 | 13.09885569 | 2.456792981 | 1.08464E-06 |
| mmu-miR-142a-5p | B vs. A | 9.708861323 | 8.514494132 | 2.288444322 | 0.000630296 |
|  | C vs. A | 9.604688958 | 8.513585375 | 2.130369359 | 0.000910438 |
|  | D vs. A | 10.66724332 | 8.57850032 | 4.25377285 | 1.68417E-13 |
| mmu-miR-7a-5p | B vs. A | 11.99837291 | 10.87025571 | 2.185733032 | 0.000132223 |
|  | C vs. A | 11.83531911 | 10.86163623 | 1.963847462 | 0.000884273 |
|  | D vs. A | 11.97603154 | 10.93965292 | 2.051072688 | 0.000192884 |
| mmu-miR-455-5p | B vs. A | 9.868949334 | 8.814865862 | 2.076398674 | 0.012040604 |
|  | C vs. A | 9.669927698 | 8.803557481 | 1.823070329 | 0.023374061 |
|  | D vs. A | 10.14611727 | 8.893186284 | 2.383251146 | 0.000353361 |
| mmu-miR-340-5p | B vs. A | 10.80558546 | 9.749942392 | 2.078644541 | 0.004025778 |
|  | C vs. A | 10.45086185 | 9.732074868 | 1.645797666 | 0.032098249 |
|  | D vs. A | 11.2256109 | 9.816011709 | 2.656633466 | 8.9932E-06 |

**Supplementary Table 2** miRNAs down-regulated.

| **microRNA** | **Group** | **B/C/D-CMP** | **A-CMP** | **Fold-Change** | **p-value** |
| --- | --- | --- | --- | --- | --- |
| mmu-miR-200a-3p | B vs. A | 3.721630223 | 5.497590648 | 0.291999858 | 3.67361E-06 |
|  | C vs. A | 9.575961562 | 11.72967158 | 0.224733949 | 1.83665E-11 |
|  | D vs. A | 4.279911262 | 6.388481003 | 0.23187678 | 9.93912E-06 |
| mmu-miR-324-5p | B vs. A | 3.047686 | 5.006276366 | 0.257279718 | 6.68809E-05 |
|  | C vs. A | 3.21523574 | 5.029838107 | 0.284282586 | 0.000346772 |
|  | D vs. A | 3.543034877 | 5.088793498 | 0.342515547 | 0.001109592 |
| mmu-miR-133b-3p | B vs. A | 8.86227304 | 10.23781537 | 0.385407798 | 0.000617769 |
|  | C vs. A | 8.602247992 | 10.26011841 | 0.316906593 | 0.000808042 |
|  | D vs. A | 8.787198096 | 10.30724049 | 0.34867567 | 6.02109E-05 |
| mmu-miR-30b-5p | B vs. A | 9.062522073 | 10.34348117 | 0.411521839 | 0.000503943 |
|  | C vs. A | 8.802394961 | 10.36293319 | 0.339024579 | 4.56242E-05 |
|  | D vs. A | 8.845032253 | 10.42080409 | 0.335463606 | 9.62689E-06 |
| mmu-miR-133a-3p | B vs. A | 11.41288232 | 12.64887117 | 0.424551405 | 0.000638139 |
|  | C vs. A | 11.32618058 | 12.66854426 | 0.394373992 | 0.002092268 |
|  | D vs. A | 11.69854097 | 12.71878966 | 0.493031356 | 0.003124145 |
| mmu-miR-101a-3p | B vs. A | 3.57878041 | 4.840692735 | 0.416990863 | 0.000369919 |
|  | C vs. A | 11.28056664 | 12.54105343 | 0.417403096 | 0.000594903 |
|  | D vs. A | 11.26011087 | 12.60422594 | 0.393895525 | 0.000120109 |
| mmu-miR-101c | B vs. A | 11.11417885 | 12.52275583 | 0.376683051 | 9.40743E-05 |
|  | C vs. A | 11.28052588 | 12.54091591 | 0.417431095 | 0.000595443 |
|  | D vs. A | 11.25995856 | 12.60408907 | 0.393891309 | 0.000120092 |
| mmu-miR-195a-5p | B vs. A | 8.277058873 | 9.797872453 | 0.348489337 | 7.35388E-06 |
|  | C vs. A | 8.555732028 | 9.811863563 | 0.418665071 | 0.000269966 |
|  | D vs. A | 8.600324248 | 9.876453921 | 0.412901718 | 8.88785E-05 |
| mmu-let-7b-5p | B vs. A | 11.45634638 | 12.32539123 | 0.547509214 | 0.002425491 |
|  | C vs. A | 11.20275314 | 12.32346165 | 0.459867929 | 7.55539E-05 |
|  | D vs. A | 10.368022 | 12.39361132 | 0.245604802 | 2.30647E-13 |
| mmu-miR-199b-5p | B vs. A | 9.611091463 | 10.72475202 | 0.462120004 | 0.002425491 |
|  | C vs. A | 9.735840235 | 10.7417151 | 0.497968068 | 7.55539E-05 |
|  | D vs. A | 9.730980628 | 10.8017774 | 0.476056011 | 2.30647E-13 |
| mmu-miR-10a-5p | B vs. A | 13.50779295 | 14.29909604 | 0.577821948 | 0.010274515 |
|  | C vs. A | 13.29539963 | 14.29616078 | 0.499736277 | 0.00053774 |
|  | D vs. A | 12.80604528 | 14.36961714 | 0.338312442 | 6.46519E-09 |
| mmu-miR-30a-5p | B vs. A | 13.32091862 | 14.19819861 | 0.544392844 | 0.003581793 |
|  | C vs. A | 13.30087512 | 14.20650221 | 0.533800628 | 0.002567644 |
|  | D vs. A | 13.0332379 | 14.27457466 | 0.422980554 | 1.39257E-05 |
| mmu-miR-26a-5p | B vs. A | 14.4803695 | 15.44104696 | 0.513815579 | 0.00324506 |
|  | C vs. A | 14.56648942 | 15.4548836 | 0.540215081 | 0.011609427 |
|  | D vs. A | 14.78949973 | 15.52199309 | 0.601862835 | 0.036201602 |
| mmu-miR-29a-3p | B vs. A | 10.48518479 | 11.37419189 | 0.539985624 | 0.009678308 |
|  | C vs. A | 10.53335197 | 11.39004068 | 0.552218562 | 0.011541695 |
|  | D vs. A | 10.61970656 | 11.45159147 | 0.561794768 | 0.010739274 |
| mmu-miR-181a-5p | B vs. A | 9.898263144 | 10.70331794 | 0.57234034 | 0.006288677 |
|  | C vs. A | 9.944358336 | 10.70119187 | 0.591793792 | 0.010151405 |
|  | D vs. A | 9.474988207 | 10.76885078 | 0.407857597 | 2.01379E-06 |
| mmu-miR-23a-3p | B vs. A | 11.32972752 | 12.3736208 | 0.48501683 | 0.000484443 |
|  | C vs. A | 11.69626877 | 12.38447224 | 0.62062621 | 0.023782887 |
|  | D vs. A | 11.17398372 | 12.44332735 | 0.414848472 | 8.19551E-06 |
